# Supplementary material for: ATR kinase supports normal proliferation in the early S phase by preventing replication resource exhaustion
Source: Nat Commun. 2023 Jun 19;14:3618. doi: 10.1038/s41467-023-39332-5 (PMC10279696; doi:10.1038/s41467-023-39332-5)

ATR kinase supports normal proliferation in the early S phase by preventing replication resource exhaustion.

Demis Menolfi[1], Brian J. Lee[1], Hanwen Zhang[1], Wenxia Jiang[1], Nicole E. Bowen[2], Yunyue Wang[1], Junfei Zhao[3], Antony Holmes[1], Steven Gershik[1], Raul Rabadan[3], Baek Kim[2], Shan Zha[1, 4, 5, 6,*]

[1] Institute for Cancer Genetics, Vagelos College for Physicians and Surgeons, Columbia University, New York City, NY 10032, USA.

[2] Department of Pediatrics, Emory University School of Medicine, Atlanta, GA 30322, USA.

[3] Program for Mathematical Genomics, Department of Systems Biology, Vagelos College for Physicians and Surgeons, Columbia University, New York City, NY 10032, USA.

[4] Department of Pathology and Cell Biology, Herbert Irvine Comprehensive Cancer Center, Vagelos College for Physicians and Surgeons, Columbia University, New York City, NY 10032, USA.

[5] Division of Pediatric Hematology, Oncology and Stem Cell Transplantation, Department of Pediatrics, Vagelos College for Physicians and Surgeons, Columbia University, New York City, NY 10032, USA.

[6] Department of Immunology and Microbiology, Vagelos College for Physicians and Surgeons, Columbia University, New York City, NY 10032, USA.

*Correspondence to: sz2296@cumc.columbia.edu

This file contains:

- Supplementary Figures
- Supplementary Figure Legends
- Uncropped blots for all the Western blots and the PCR agarose gels of the paper

Supplementary Figure 1

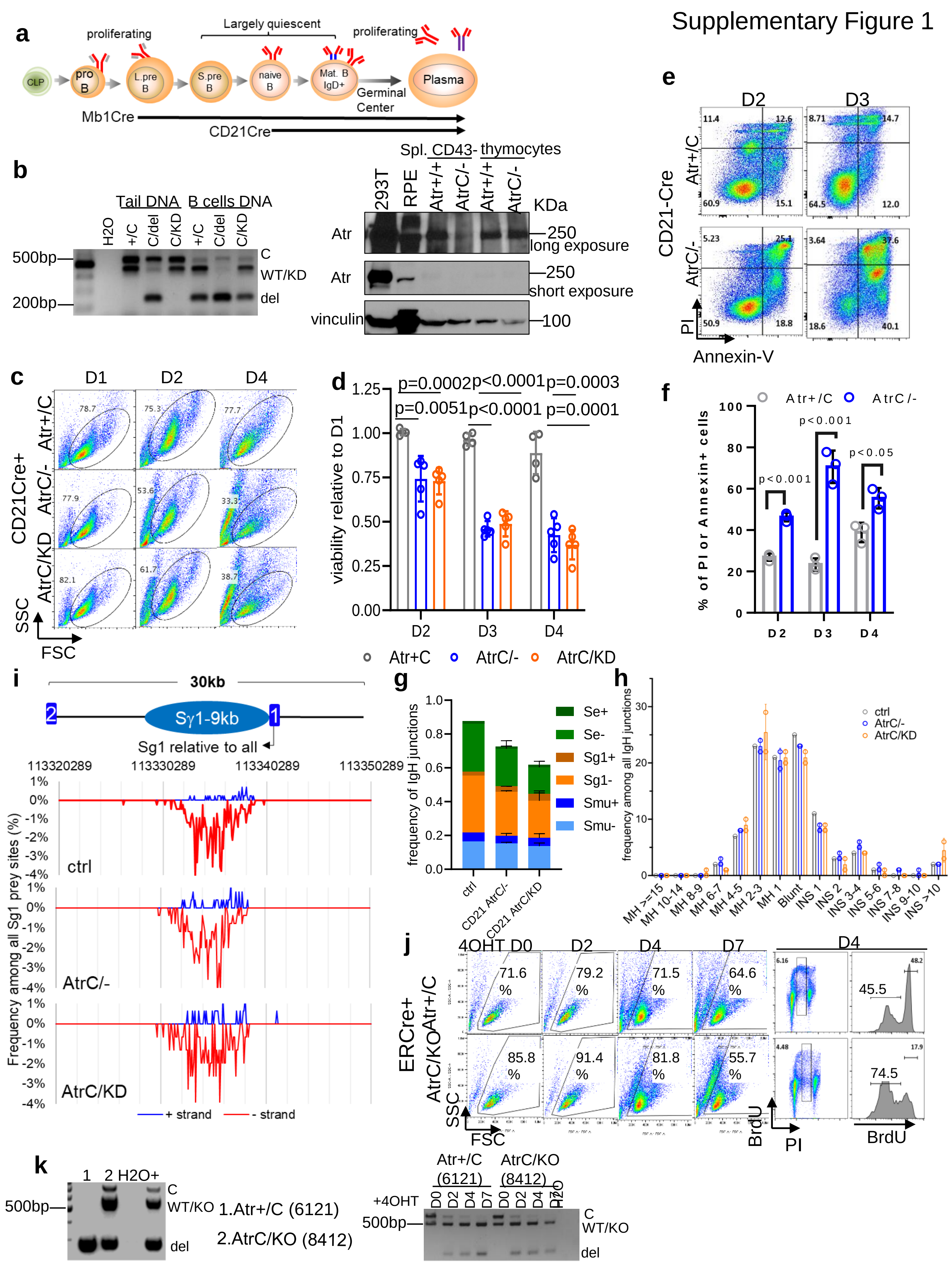

Supplementary Figure 1

a) Schematic representation of B cell development and B cell sub-types, with Mb1 and CD21 time of expression activation indicated. b) PCR and Western Blot analyses of ATR in CD21Cre+ B and T cells. For the PCR, DNA was collected from mice tails and CD43- splenic B cells of the indicated genotypes. A three-primer PCR that separately identifies the conditional C (530 bp), the WT/KD (450 bp), and the deleted (240 bp) *Atr* alleles was performed. H2O was used as a negative control. For the Western Blotting, total proteins were collected from control human cell lines and CD43- splenic cells (~90%+ B cells) and thymocytes of the indicated genotypes. c) Examples of flow cytometry profiles of *CD21-Cre*$^{+}$ *Atr*$^{+/C}$, *Atr*$^{C/-}$ and *Atr*$^{C/KD}$ cell viability at D1, D2, and D4 post-stimulation. d) Quantification of cell viability relative to D1 from four or five biologically independent experiments. Data are presented as mean values +/- SEM. e) Flow cytometry analyses with apoptosis markers (Annexin-V and PI) in activated *CD21-Cre*$^{+}$ *Atr*$^{+/C}$ and *Atr*$^{C/-}$ B cells. f) Quantification of the apoptotic cells (Annexin V or PI positive). An unpaired two-tailed t-test was used in d and f. g). The frequency of IgH junctions recovered for the positive and negative strands of Sε, Sγ, and Sμ is reported for one control, two *CD21-Cre*$^{+}$ *Atr*$^{C/-}$ and two *Atr*$^{C/KD}$ biologically independent mice. Data are presented as mean values +/- SEM. h) The frequency of blunt ends, microhomology (MH), and insertions (INS) are reported for one control, two *CD21-Cre*$^{+}$ *Atr*$^{C/-}$ and two *Atr*$^{C/KD}$ biologically independent mice. Data are presented as mean values +/- SEM. i) Schematic representation of the Sγ1 region analyzed in HTGTS experiments. Junctions sequenced are reported for ctrl, *CD21-Cre*$^{+}$ *Atr*$^{C/-,}$ and *Atr*$^{C/KD}$. j) Flow cytometry analyses of viability (FSC/SSC) and by Annexin-V and PI in v-abl kinase transformed B cells. k) Genotyping of the *Rosa-ER-Cre*$^{+}$ *Atr*$^{+/C}$ or *Atr*$^{C/KO}$ cell lines before and after 4 hydroxy-tamoxifen (4OHT). The AtrKO and Atr del (from C) are two alleles of Atr null. See the methods section for details.

Source Data are provided as a Source Data file.

Supplementary Figure 2

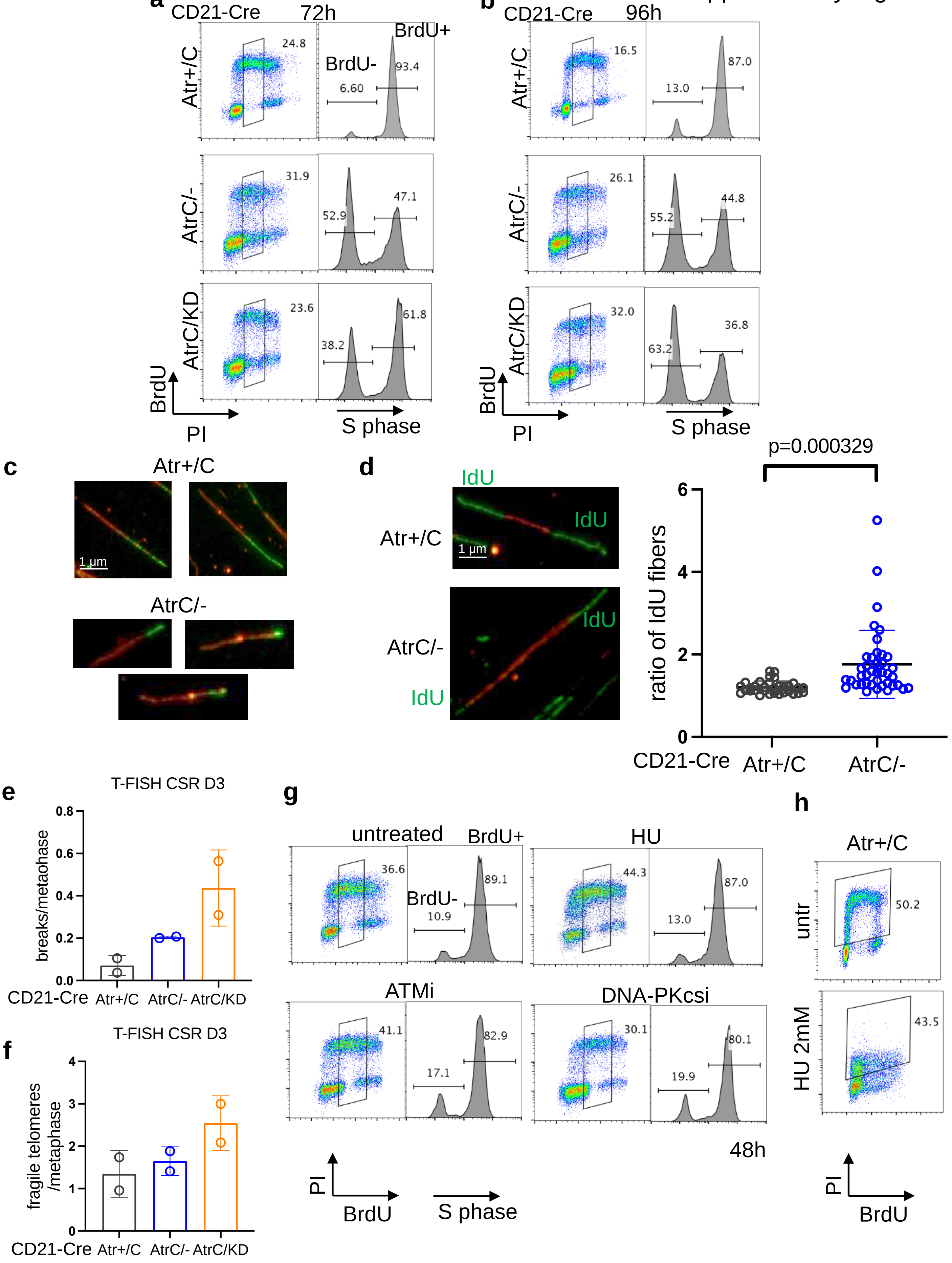

Supplementary Figure 2

a) b) Flow cytometry profiles of *CD21-Cre*$^{+}$ *Atr*$^{+/C}$, *Atr*$^{C/-,}$ and *Atr*$^{C/KD}$ B cells pulse-labeled with BrdU for 30 minutes at 72 h or 96 h post-stimulation. Representative dot plots are shown. Histogram plots of S phase cells are shown with the separation in BrdU-negative and BrdU-positive cells. c) Representative DNA fibers for the experiment reported in Figure 2b. d) DNA replication fork symmetry was calculated for CD21-Cre *Atr*$^{+/C}$ and *Atr*$^{C/-}$ B cells. Representative images are shown, and quantification of the ratio between two IdU forks originating from the same CldU track is shown from one representative of two independent experiments is shown. Two-tailed t test was used for the statistical analysis. e) f) T-FISH analysis was performed on *CD21-Cre*$^{+}$ *Atr*$^{+/C}$, *Atr*$^{C/-,}$ and *Atr*$^{C/KD}$ B cells at D3 post-stimulation collected from two biologically independent mice per each genotype. The mean number and standard error of breaks and fragile telomeres per metaphase are shown for each genotype analyzed. g) 48h stimulated WT B cells were left untreated, treated with 20 µM HU, 7.5 µM ATMi (KU-55933), or 2.5 µM DNA-PKcsi (NU7441) for the length of the experiment. Dot plots and corresponding histograms of S phase cells with BrdU-negative and BrdU-positive separation are shown. h) Control *CD21-Cre*$^{+}$ *Atr*$^{+/C}$ cells were treated with 2 mM HU for 24 hours from stimulation. BrdU was added for 30 minutes before collection.

Source Data are provided as a Source Data file.

Supplementary Figure 3

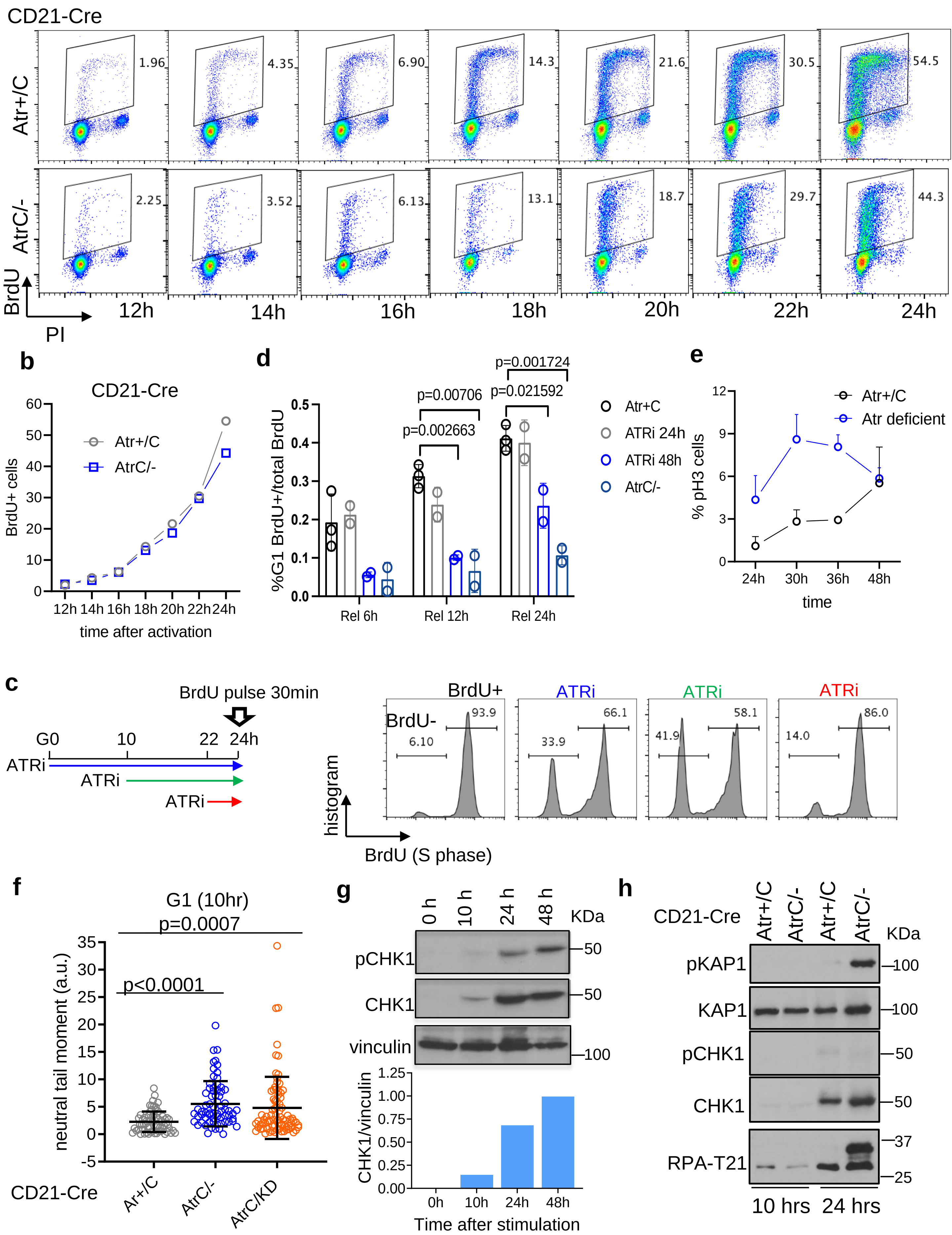

Supplementary Figure 3

a) *CD21-Cre*$^{+}$ *Atr*$^{+/C}$ and *Atr*$^{C/-}$ B cells were stimulated for CSR, and 12 hours after stimulation, pulse-labeled with BrdU for 30 minutes every 2 h until 24 h. Dot plots of BrdU/PI are reported with quantification of S phase cells for every time point. b) S phase BrdU-positive cells from the experiment in (a) are plotted. c) 24 h stimulated WT B cells were left untreated or treated with 5 µM ATRi (VE-821) for 24 h, 14 h, or 2 h. Cells were pulse-labeled with BrdU for 30 minutes before collection and double-stained for BrdU and PI. Histograms of S phase cells with BrdU-negative and BrdU-positive separation are shown. d) Quantification of two or three independent experiments reported in Figure 3e. Statistical analysis was performed using an unpaired two tail t-test. e) Quantification of two biologically independent samples per line for the experiment shown in Figure 3f. The percentage of pH3+ cells is reported for every time point, and overlays of different genotypes are shown as indicated. Data are presented as mean values +/- SEM. f) *CD21-Cre*$^{+}$ *Atr*$^{+/C}$, *Atr*$^{C/-}$ and *Atr*$^{C/KD}$ B cells were collected for neutral comet assay at 10 h post-stimulation (G1 time point). The tail moment is reported in arbitrary units (a.u.), and statistical analysis has been performed using the two-tailed Mann-Whitney test. Data are presented as mean values +/- SD. g) Naïve B cells were purified from a WT mouse and stimulated for CSR. Cells were collected at 0 h, 10 h, 24 h, and 48 h post-stimulation and lysed for protein analysis. Western Blot of pCHK1 S345, total CHK1, and vinculin were performed. Quantification of the ratio of total CHK1/vinculin is reported. h) *CD21-Cre*$^{+}$ *Atr*$^{+/C}$ and *Atr*$^{C/-}$ B cells were collected and lysed for protein analysis at 10 and 24 h post-stimulation. Western Blot was performed for the proteins indicated.

Source Data are provided as a Source Data file.

Supplementary Figure 4

a

CD21-Cre Atr+/C, G0 to G1 upregulated genes

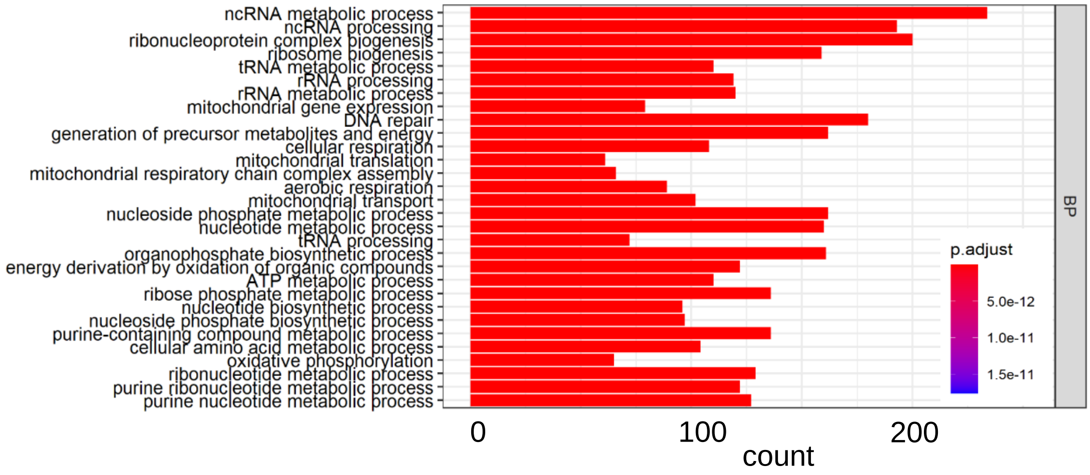

CD21-Cre AtrC/-, G0 to G1 upregulated genes

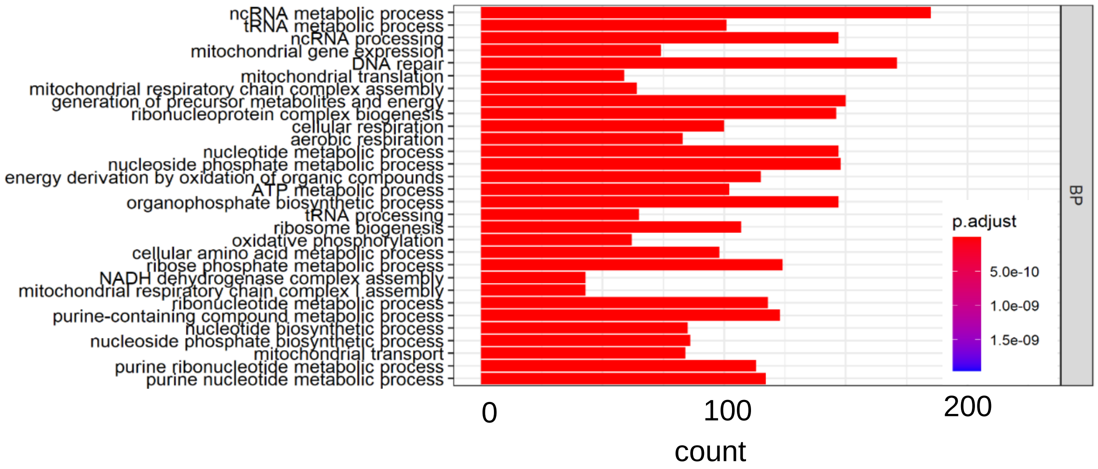

b

DNA replication initiation

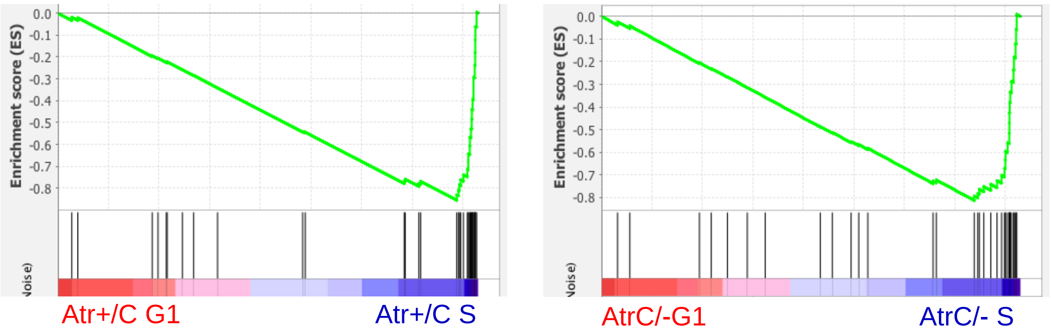

Supplementary Figure 4

a) Gene ontology (GO) analysis was performed on genes that are significantly upregulated from the G0 to G1 phase with a fold increase (FC) of at least 1.5 (log2>0.585) and a padjust<0.01. Samples were analyzed from RNA-seq, as in Figure 4a. b) Gene Set Enrichment Analysis (GSEA) was performed for DNA replication initiation genes, comparing the increased expression from G1 to S phase for both *CD21-Cre*$^{+}$ *Atr*$^{+/C}$ and *Atr*$^{C/-}$ cells.

Source Data are provided as a Source Data file.

Supplementary Figure 5

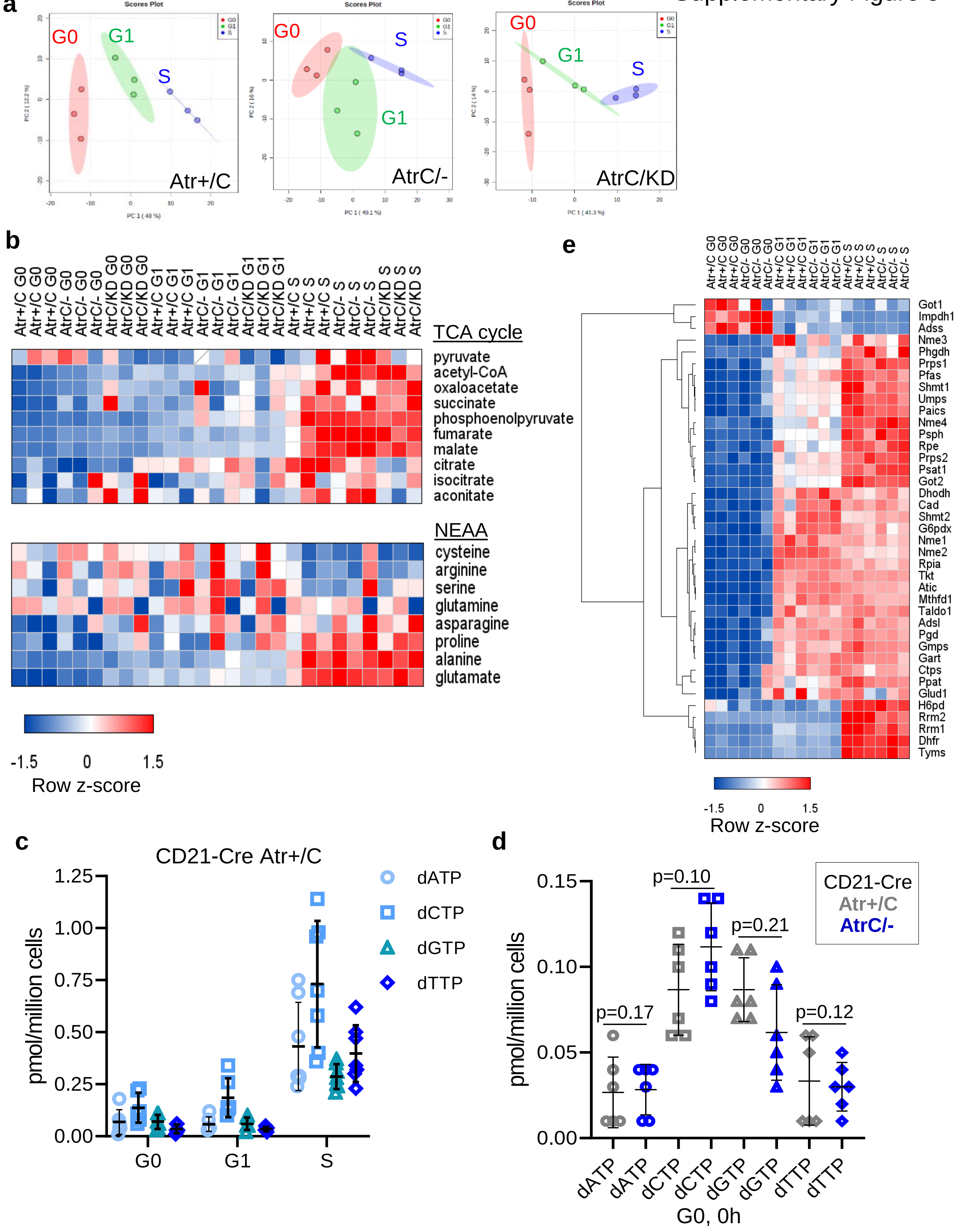

Supplementary Figure 5

a) Principal component analysis (PCA) of metabolite distribution in G0, G1, and S phase for *CD21-Cre*$^{+}$ *Atr*$^{+/C}$, *Atr*$^{C/-}$ and *Atr*$^{C/KD}$ B cells. b) Heatmaps of z-scores of integrated peak area values of metabolites of the TCA cycle and non-essential amino acids for *CD21-Cre*$^{+}$ *Atr*$^{+/C}$, *Atr*$^{C/-}$ and *Atr*$^{C/KD}$ in G0, G1, and S phase. c) dNTP (dATP, dCTP, dGTP, and dTTP) amounts in pmol/million cells were plotted for control cells in G0, G1, and S phases derived from six or seven biologically independent samples. Data are presented as mean values +/- SEM. d) dNTP (dATP, dCTP, dGTP, and dTTP) amounts in pmol/million cells were quantified from six *CD21-Cre*$^{+}$ *Atr*$^{+/C}$ and six *Atr*$^{C/-}$ B cell samples, collected from independent mice, at the time of activation (G0). Statistical analysis was performed using a two-tailed t-test. Data are presented as mean values +/- SEM. e) Heatmap of the z-scores of tpm values of the enzymes of nucleotide biosynthesis (purines, pyrimidines, ribose/PPP, and feeder pathways) obtained from RNA-seq of *CD21-Cre*$^{+}$ *Atr*$^{+/C}$ and *Atr*$^{C/-}$ cells in G0, G1, and S phase. The clustering of rows was done using Pearson distance and complete linkage.
Source Data are provided as a Source Data file.

Supplementary Figure 6

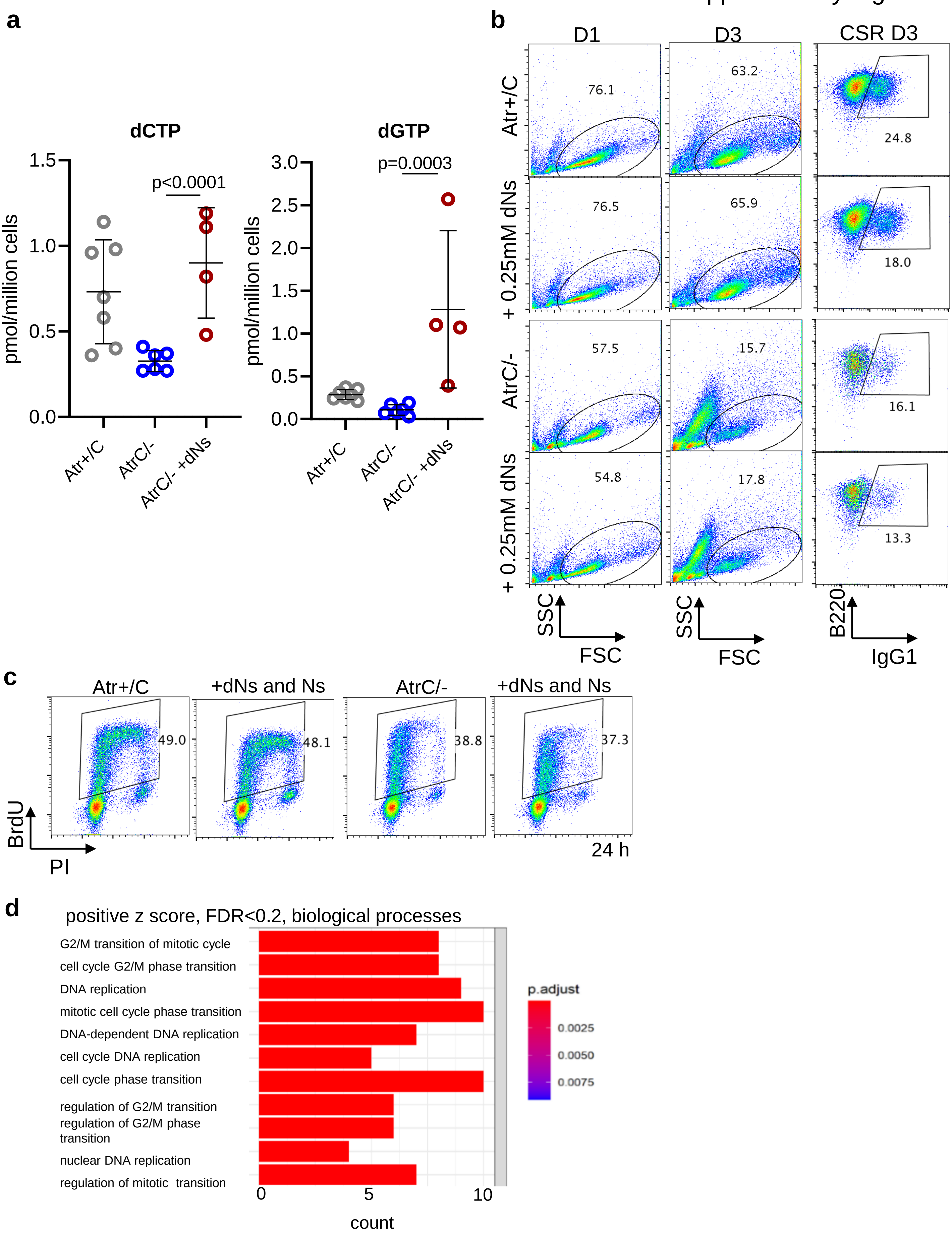

Supplementary Figure 6

a) Quantifications of dCTP and dGTP, as described in Figure 6a are reported. Two-tailed t test was used. b) *CD21-Cre*$^{+}$ *Atr*$^{+/C}$ and *Atr*$^{C/-}$ B cells untreated or supplemented with 0.25 mM of deoxyribonucleosides were analyzed for viability and CSR efficiency. Viability is reported for D1 and D3. CSR efficiency at D3 is shown as a percentage of IgG1-B220 positive cells by flow cytometry. c) *CD21-Cre*$^{+}$ *Atr*$^{+/C}$ and *Atr*$^{C/-}$ B cells untreated or supplemented with 0.05 mM of deoxyribonucleosides and 1X Embryomax (nucleosides) were analyzed for BrdU incorporation at 24 h post-stimulation. d) Genes with positive z-score and FDR<0.2 identified in the CRISPR screen were analyzed for Gene Ontology. Significant biological processes are reported.

Source Data are provided as a Source Data file.

Supplementary Figure 7

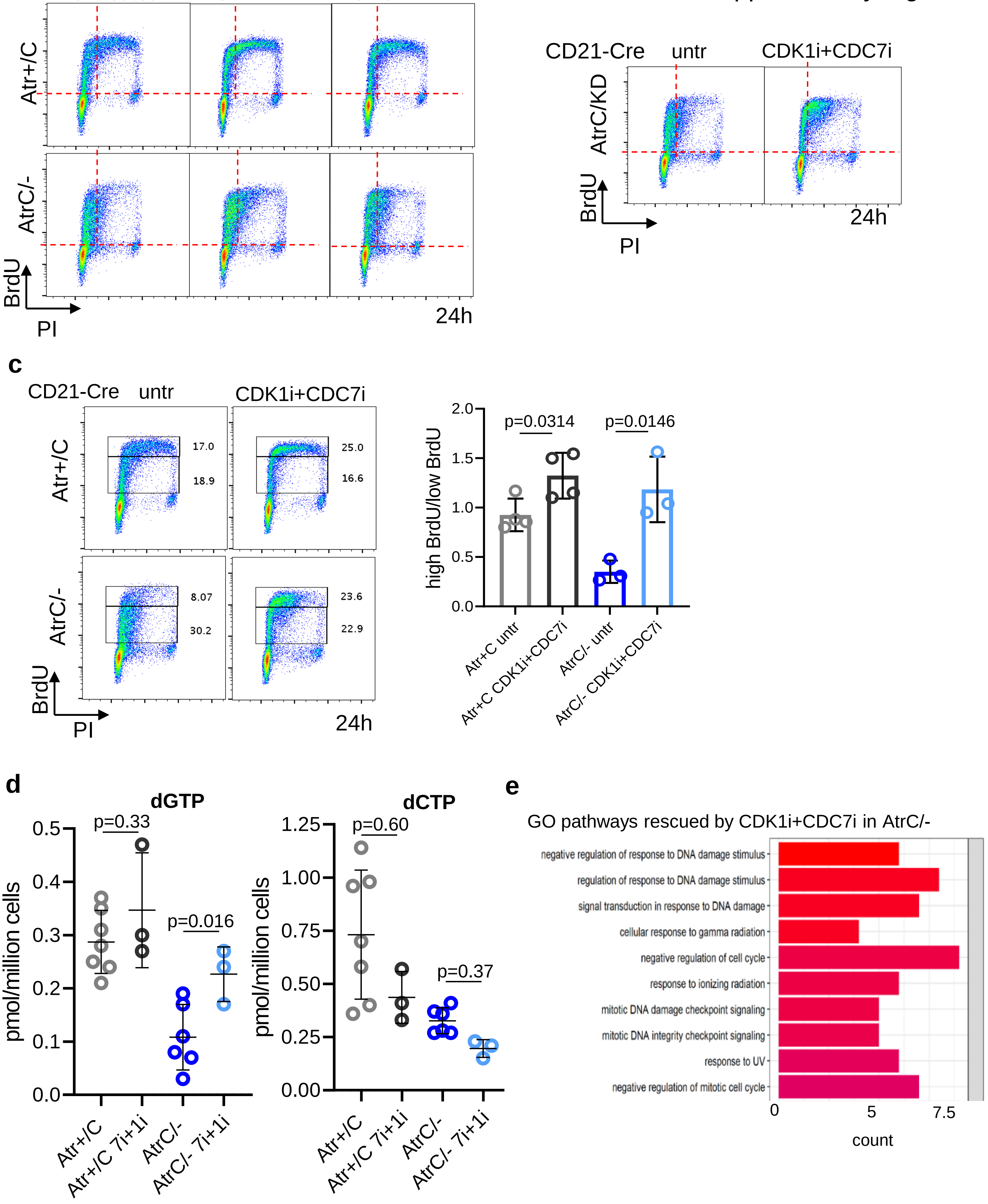

Supplementary Figure 7

a) *CD21-Cre*$^{+}$ *Atr*$^{+/C}$ and *Atr*$^{C/-}$ cells untreated, or treated at 10 h post-stimulation with 2.5 µM of CDC7i (XL413) or 2.5 µM of CDK1i (Ro-3306), were pulse-labeled with BrdU for 30 minutes and collected at 24 h. Cell-cycle profiles are shown. b) *CD21-Cre*$^{+}$ *Atr*$^{C/KD}$ cells were left untreated or treated at 10h post-stimulation with 2.5 µM of CDC7i + 2.5 µM of CDK1i for 14 h and were pulse-labeled with BrdU for 30 minutes and collected at 24h. c) Experiments, done as in Figure 7a, were analyzed for the ratio between high BrdU vs low BrdU as shown in the representative flow cytometry profiles. Statistical analysis was performed using two tailed t test. d) Quantifications of dCTP and dGTP as described in Figure 7g are reported. e) Gene ontology analysis of genes significantly upregulated in *CD21-Cre*$^{+}$ *Atr*$^{C/-}$ cells in S phase compared to control *CD21-Cre*$^{+}$ *Atr*$^{+/C}$ cells (FC>1.5, padjust<0.01), but rescued by CDC7i+CDK1i treatment.

Source Data are provided as a Source Data file.

Uncropped western blots and PCR gels

Figure 2e

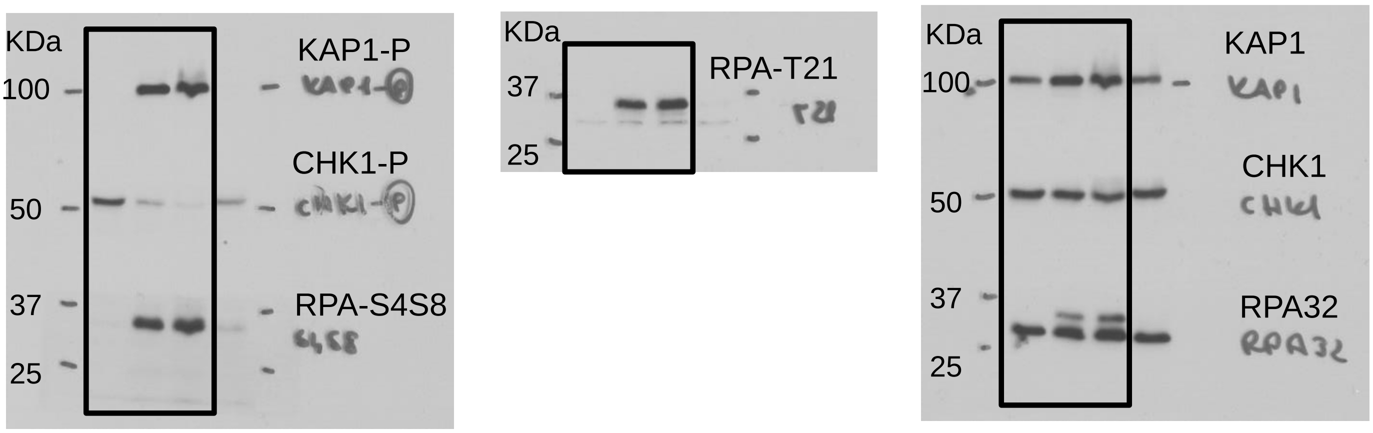

Figure 6c

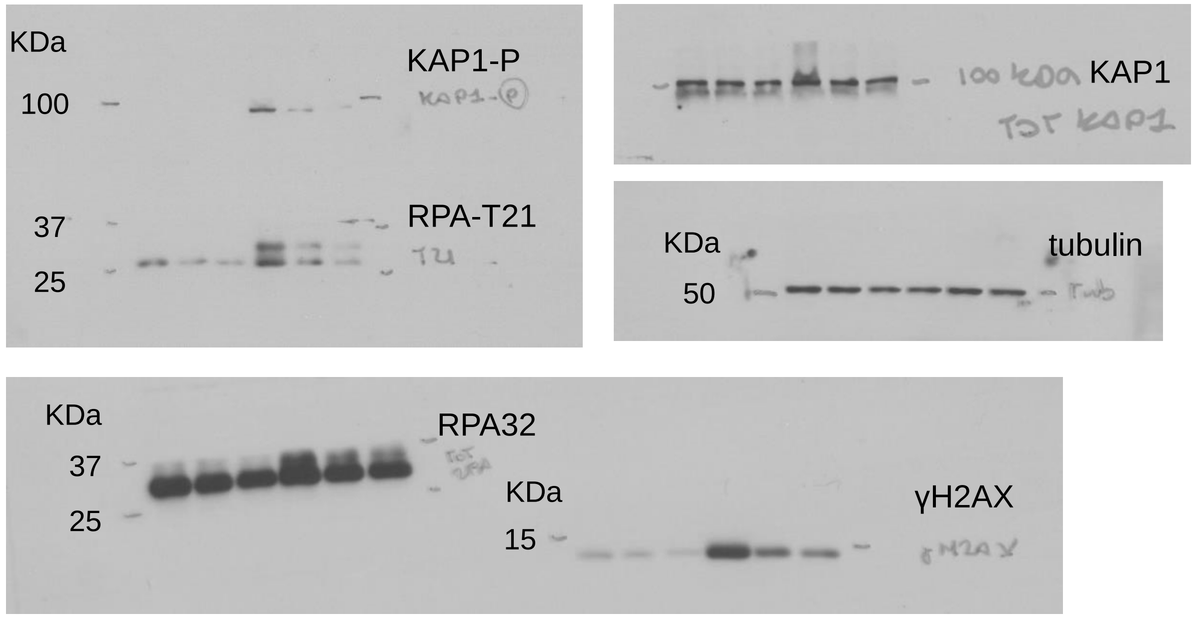

Figure 7d

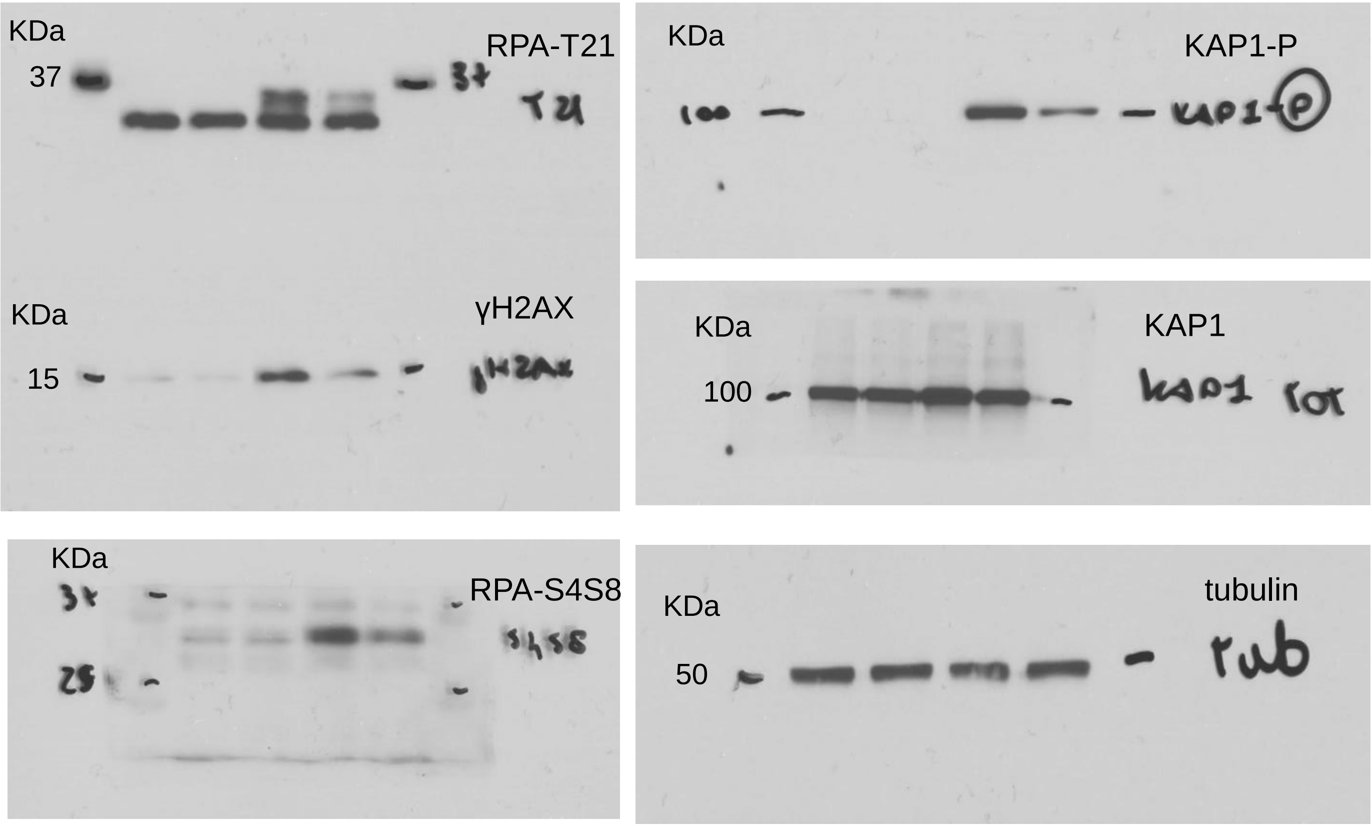

Figure S1b

PCR gel

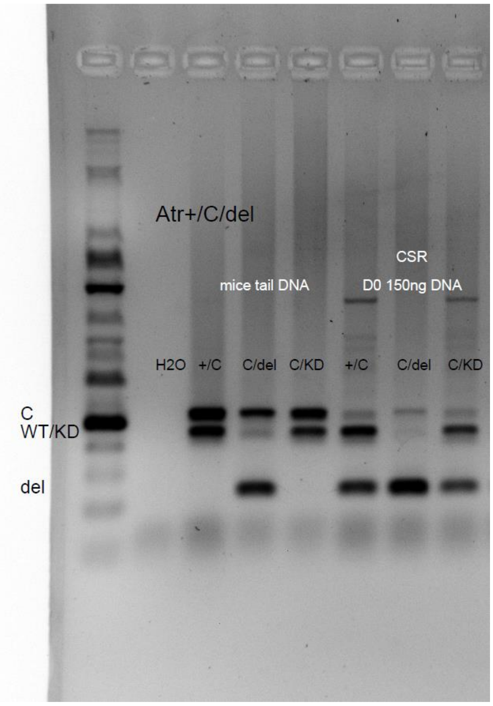

Westen blot

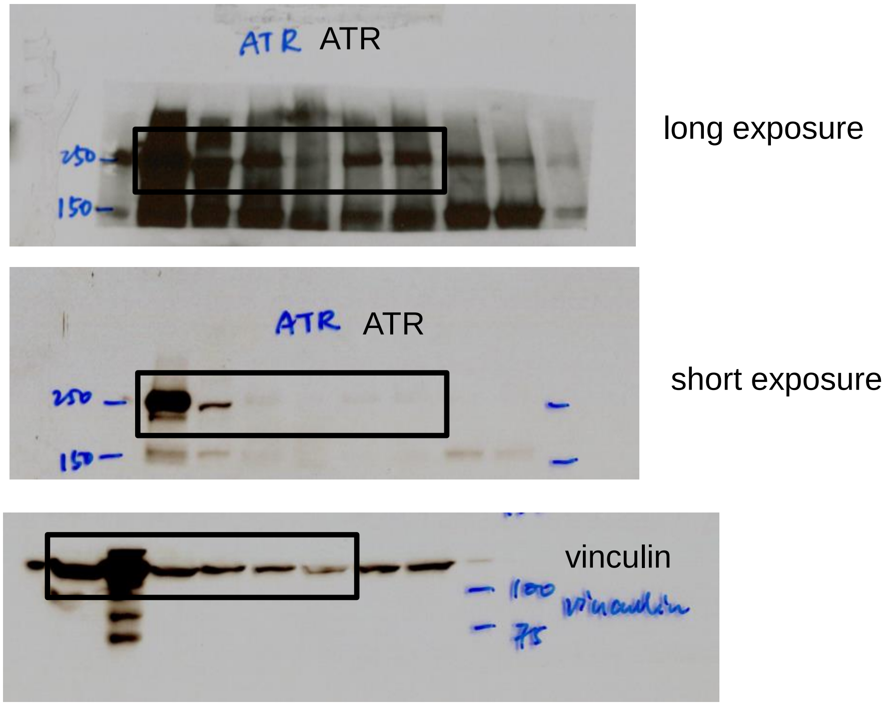

Figure S1k

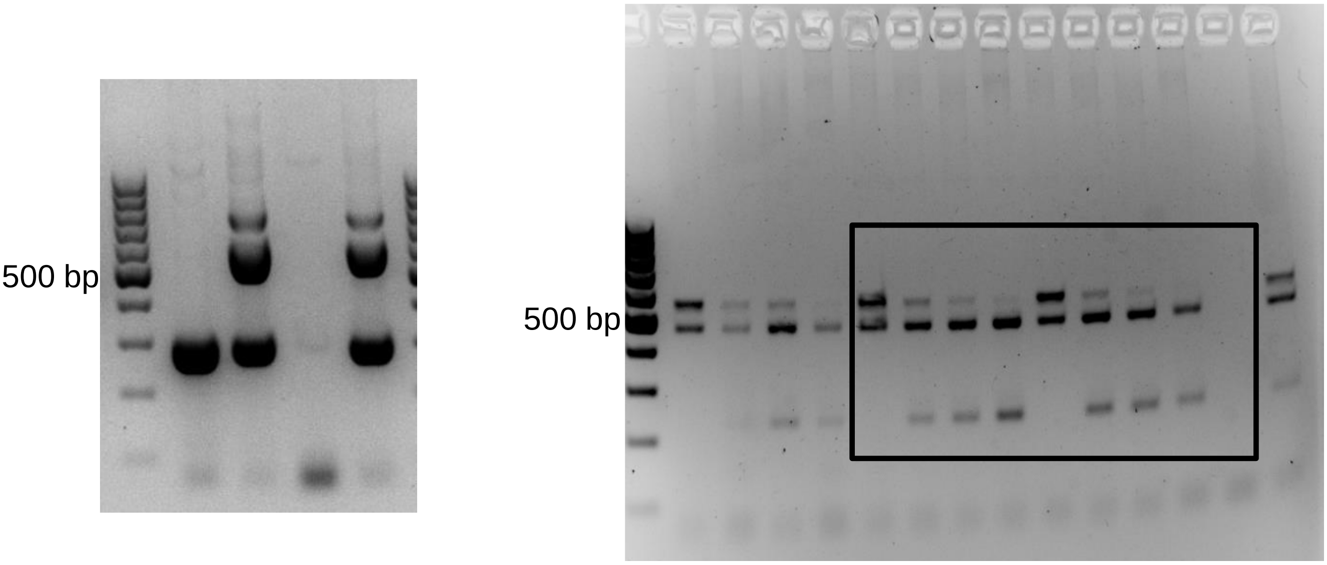

Figure S3g

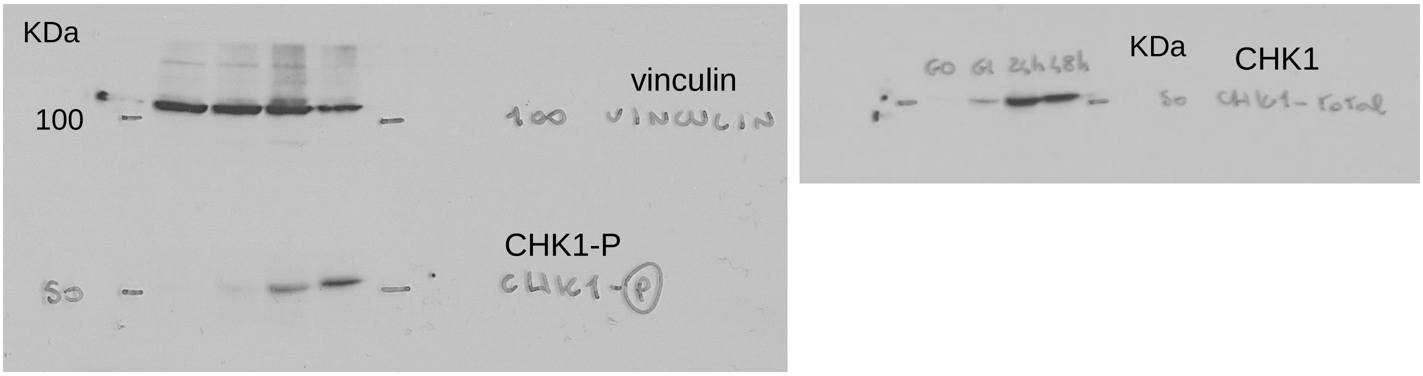

Figure S3h

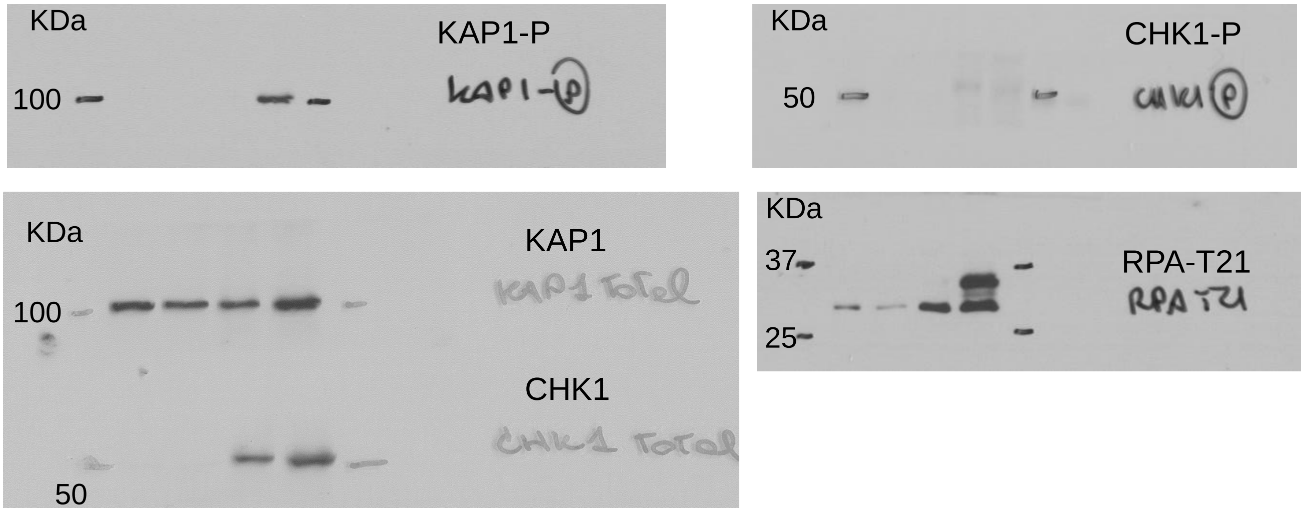

Supplement: Supplementary file 1 — Supplementary Information [file 41467_2023_39332_MOESM1_ESM.pdf]
