# Supplementary material for: ATR kinase supports normal proliferation in the early S phase by preventing replication resource exhaustion
Source: Nat Commun. 2023 Jun 19;14:3618. doi: 10.1038/s41467-023-39332-5 (PMC10279696; doi:10.1038/s41467-023-39332-5)
Supplement: Supplementary file 3 — Description of Additional Supplementary Files [file 41467_2023_39332_MOESM3_ESM.pdf]

Description of Additional Supplementary Files

File Name: Supplementary Data 1
Description: List of the gRNA targets that are consistently enriched upon ATR inhibitor and CHK1 inhibitor treatment. The CRISPR data were analzyed via the MAGeCK package and the FDR and Z score are direct output from the MAGeCK pipeline. The data includes the beta score, z score, p value and FDR for each target. 57 targets were found to be enriched (positive z score) in at least two of the four screens (2 ATRi VE-821, 1 ATRi AZD6738, and 1 CHK1i LY2603618) with an FDR.
