# Supplementary material for: ATR kinase supports normal proliferation in the early S phase by preventing replication resource exhaustion
Source: Nat Commun. 2023 Jun 19;14:3618. doi: 10.1038/s41467-023-39332-5 (PMC10279696; doi:10.1038/s41467-023-39332-5)
Supplement: Supplementary file 5 — Reporting Summary [file 41467_2023_39332_MOESM5_ESM.pdf]

nature portfolio

Corresponding author(s): Zha Shan

Last updated by author(s): May 19, 2023

Reporting Summary

Nature Portfolio wishes to improve the reproducibility of the work that we publish. This form provides structure for consistency and transparency in reporting. For further information on Nature Portfolio policies, see our Editorial Policies and the Editorial Policy Checklist.

Statistics

For all statistical analyses, confirm that the following items are present in the figure legend, table legend, main text, or Methods section.

n/a	Confirmed	
☐	☒	The exact sample size (*n*) for each experimental group/condition, given as a discrete number and unit of measurement
☐	☒	A statement on whether measurements were taken from distinct samples or whether the same sample was measured repeatedly
☐	☒	The statistical test(s) used AND whether they are one- or two-sided<br>*Only common tests should be described solely by name; describe more complex techniques in the Methods section.*
☒	☐	A description of all covariates tested
☒	☐	A description of any assumptions or corrections, such as tests of normality and adjustment for multiple comparisons
☐	☒	A full description of the statistical parameters including central tendency (e.g. means) or other basic estimates (e.g. regression coefficient) AND variation (e.g. standard deviation) or associated estimates of uncertainty (e.g. confidence intervals)
☐	☒	For null hypothesis testing, the test statistic (e.g. *F*, *t*, *r*) with confidence intervals, effect sizes, degrees of freedom and *P* value noted<br>*Give P values as exact values whenever suitable.*
☒	☐	For Bayesian analysis, information on the choice of priors and Markov chain Monte Carlo settings
☐	☒	For hierarchical and complex designs, identification of the appropriate level for tests and full reporting of outcomes
☐	☒	Estimates of effect sizes (e.g. Cohen's *d*, Pearson's *r*), indicating how they were calculated

Our web collection on statistics for biologists contains articles on many of the points above.

Software and code

Policy information about availability of computer code

Data collection	CellQuest Pro on FACScalibur (Becton Dickinson)<br>Attune NxT Software v4.2.0 on Attune NxT Acoustic Focusing Cytometer (Invitrogen)<br>ISIS fuorescence imaging (MetaSystems) on Carl Zeiss AxioImager Z2 microscope<br>NIS-Elements AR software on Nikon 80i fluorescence microscope (Nikon, Inc)<br>NovaSeq 6000 (Illumina)<br>MiSeq 500v (Illumina)<br>NextSeq 550 machine (Illumina)<br>PharosFX molecular imager (Bio-Rad)
Data analysis	GraphPad Prism 9<br>Microsoft Excel Windows 10<br>FlowJo X<br>Isis fluorescence imaging platform (MataSystems)<br>CometScore version 1.5<br>ImageJ (Image processing and analysis in Java)<br>Metaboanalyst 5.0<br>Illumina RTA version 1.18.54<br>bcl2fastq2 (version 2.20)<br>Bowtie2<br>R i386 4.1.2<br>R package ClusterProfiler

R package Sleuth
MAGeCK FluteMLE pipeline
Image Lab software, version 5.1.2, (Bio-Rad)
Kallisto 0.44.0

For manuscripts utilizing custom algorithms or software that are central to the research but not yet described in published literature, software must be made available to editors and reviewers. We strongly encourage code deposition in a community repository (e.g. GitHub). See the Nature Portfolio guidelines for submitting code & software for further information.

Data

Policy information about availability of data

All manuscripts must include a data availability statement. This statement should provide the following information, where applicable:

- Accession codes, unique identifiers, or web links for publicly available datasets
- A description of any restrictions on data availability
- For clinical datasets or third party data, please ensure that the statement adheres to our policy

The authors declare that the data supporting the findings of this study are available with the paper and its supplementary information files. The RNA-sequencing, HTGTS and CRISPR-Cas9 screen data generated in this study have been deposited in the GEO database under accession codes GSE212194 (https://www.ncbi.nlm.nih.gov/geo/query/acc.cgi?acc=GSE212194), GSE212195 (https://www.ncbi.nlm.nih.gov/geo/query/acc.cgi?acc=GSE212195), GSE212196 (https://www.ncbi.nlm.nih.gov/geo/query/acc.cgi?acc=GSE212196) and GSE214643 (https://www.ncbi.nlm.nih.gov/geo/query/acc.cgi?acc=GSE214643), which are publicy available. All the data presented in graphs within the figures generated in this study are provided in the Source Data excel file. The uncropped gels and western blots are provided in the Supplementary Information. All data are available from the authors upon request

Human research participants

Policy information about studies involving human research participants and Sex and Gender in Research.

Reporting on sex and gender	N/A
Population characteristics	N/A
Recruitment	N/A
Ethics oversight	N/A

Note that full information on the approval of the study protocol must also be provided in the manuscript.

Field-specific reporting

Please select the one below that is the best fit for your research. If you are not sure, read the appropriate sections before making your selection.

☒ Life sciences ☐ Behavioural & social sciences ☐ Ecological, evolutionary & environmental sciences

For a reference copy of the document with all sections, see nature.com/documents/nr-reporting-summary-flat.pdf

Life sciences study design

All studies must disclose on these points even when the disclosure is negative.

Sample size	Sample size was determined according to common standard in the field. Several (n>=3) independent mice and independent primary cell (B cells) of each genotype were analyzed for each phenotype described. The number of independent samples (mice and primary B cells) is reported in the figure legends
Data exclusions	All valid experimental data were included in the analyses.
Replication	All experiments presented here were validated in independent experiments and/or with independently derived primary B cells or animals. Experiments were independently performed at least twice, with most of the experiments three or more times (the number of independent experiments is reported in all relevant figure legends).
Randomization	Mice were chosen based on their genotype, independently of their gender. Appropriate controls were included in all experiments.
Blinding	Experiments were not performed blindly. However, the researchers carried out all experiments and analyses without any prior biases and in an objective, rigorous scientific way.

Reporting for specific materials, systems and methods

We require information from authors about some types of materials, experimental systems and methods used in many studies. Here, indicate whether each material, system or method listed is relevant to your study. If you are not sure if a list item applies to your research, read the appropriate section before selecting a response.

Materials & experimental systems

n/a	Involved in the study	
☐	☒	Antibodies
☐	☒	Eukaryotic cell lines
☒	☐	Palaeontology and archaeology
☐	☒	Animals and other organisms
☒	☐	Clinical data
☒	☐	Dual use research of concern

Methods

n/a	Involved in the study	
☒	☐	ChIP-seq
☐	☒	Flow cytometry
☒	☐	MRI-based neuroimaging

Antibodies

Antibodies used

Western Blot:
rabbit polyclonal pKAP1 (S824), A300-767A, Bethyl Laboratories
rabbit monoclonal KAP1 (TIF1β), C42G12, Cell Signaling, #4124
rabbit monoclonal pCHK1 Ser245 (133D3), Cell Signaling, #2348
mouse monoclonal CHK1 (2G1D5), Cell Signaling, #2360
rabbit monoclonal anti-RPA32/RPA2 (phospho T21), Abcam, ab109394
rabbit polyclonal phospho RPA32 (S4/S8), A300-245A, Bethyl Laboratories
rabbit polyclonal RPA32 antibody, A300-244A, Bethyl Laboratories
rabbit polyclonal anti phospho H2AX (Ser139), 07-164, Millipore
mouse monoclonal anti-vinculin antibody, clone V284, 05-386, Millipore
mouse monoclonal anti α-tubulin (DM1A), CP06, Millipore
rabbit polyclonal ATR antibody, 2790, Cell Signaling

Immunofluorescence:
rat monoclonal anti-BrdU antibody [BU1/75 (ICR1)], Abcam, ab6326
mouse anti-BrdU, clone B44, Beckton Dickinson, cat. No. 347580
goat anti-mouse Alexa 488 Thermo Fisher Scientific, A-11001
goat anti-rat Alexa 594 Thermo Fisher Scientific, A-11007

FACS analysis:
FITC-conjugated mouse anti-BrdU antibody, BD Pharmingen, cat. No. 556028
Serine 10 pH3 antibody (Millipore, 06-570)
H2AX Ser139 20E3 (Cell Signaling #9718, 1:500)
FITC rat anti-mouse IgG1, clone A85-1, BD Pharmingen, cat. No. 553443
PE-Cyanine5 rat anti-mouse CD45R/B220, clone RA3-6B2, BD Pharmingen, cat. No. 553091
Goat anti-mouse IgM, Human ads-PE, SouthernBiotech, cat. No. 1020-09
FITC anti-mouse CD43 (Biolegend, 553270)
FITC-Annexin V (BD Pharmingen, Cat.No. 556419)
FITC anti-mouse/human GL7 Antigen (T and B cell Activation Marker) Antibody (Biolegend, Cat.No. 144604)
PE Hamster Anti-Mouse CD95 (BD, Cat.No. 561985)
goat anti-rabbit Alexa 488 Thermo Fisher Scientific, A-11008

Immunohistochemistry
FITC anti-mouse/human GL7, Biolegend, 144604
PE hamster anti-mouse CD95, BD, 561985

Validation

All antibodies described and used in this study have been extensively validated by the manufacturers (see manufacturer web site for details) and in our control experiments (including negative control as well as control animals or cells that show expected distribution of immune cell populations).

Eukaryotic cell lines

Policy information about cell lines and Sex and Gender in Research

Cell line source(s)

v-abl kinase transformed immature murine B cell lines generated using the bone marrow recovered from the femurs of 3-4 weeks old mice of the indicated genotypes. RPE1 cells were gifted by Dr. Keith Caldecott and were originally from ATCC. The 293T cells were originally purchased from ATCC.

Authentication

genotype validated (included in the Supplementary figures)

Mycoplasma contamination	not detected
Commonly misidentified lines (See ICLAC register)	N/A

Animals and other research organisms

Policy information about studies involving animals; ARRIVE guidelines recommended for reporting animal research, and Sex and Gender in Research

Laboratory animals	Mice (Mus Musculus) were used in this study, either in 129/Sv, C57BL/6 or mixed background. Mice were between 6-12 weeks of age for development analyses and at about 30 weeks of age for germinal center response experiments (without immunization). Mice were housed in a pathogen-free facility at the Institute of Cancer Genetics at Columbia University Medical Center, according to the Institutional Animal Care and Use Committee (IACUC) of Columbia University. Standard conditions of dark/light cycle, ambient temperature and humidity were used as provided by IACUC. Mice with the Mb1Cre (CD79) knockin allele, transgenic Cd21-Cre allele and the Rosa-ER-Cre allele were used.
Wild animals	This study did not involve wild animals
Reporting on sex	Mice development and germinal center analyses were performed equally on both male and female mice. Splenic purified B cells were equally collected for in vitro cell culture from male or female mice.
Field-collected samples	This study did not involve samples collected from the field
Ethics oversight	All animal work was conducted in the specific pathogen-free facility in Columbia University Medical Center and was approved by the Institutional Animal Care and Use Committee (IACUC) of Columbia University

Note that full information on the approval of the study protocol must also be provided in the manuscript.

Flow Cytometry

Plots

Confirm that:

☒ The axis labels state the marker and fluorochrome used (e.g. CD4-FITC).

☒ The axis scales are clearly visible. Include numbers along axes only for bottom left plot of group (a 'group' is an analysis of identical markers).

☒ All plots are contour plots with outliers or pseudocolor plots.

☒ A numerical value for number of cells or percentage (with statistics) is provided.

Methodology

Sample preparation	Sample preparation is extensively described in Material and Methods. For lymphocyte development studies, mice cells are recovered from spleen or bone marrow and are resuspended in PBS 1X and kept on ice before staining with indicated antibodies. For cell cycle analysis, primary B cells purified from the mice spleen were culture in RPMI medium supplemented with cytokines (anti-CD40 and IL-4). At the days indicated B cells were collected, washed in 1X PBS and stained with IgG1 and B220 antibody for Class Switch Recombination (CSR) analysis. For cell cycle analysis, at the time points indicated, B cells were washed in PBS 1X, fixed in EtOH 70% at 4C and subsequently stained with BrdU/PI and, occasionally, pH3 antibodies. Samples were then processed as indicated in the Material and Methods section.
Instrument	FACScalibur flow cytometer (BD Biosciences) and Attune NxT Acoustic Focusing Cytometer (Invitrogen)
Software	CellQuest Pro software for data collection on FACScalibur and Attune NxT Software v4.2.0 on Attune NxT Acoustic Focusing Cytometer (Invitrogen). FlowJo X software for data analysis.
Cell population abundance	For development analyses, primary and secondary lymphoid tissues were directly isolated for FACS analyses without purifications. For CSR analyses, splenic B cells were purified to exclude CD43+ (most T cells and myeloid cells) cells. The purified splenic B cell population were routinely analyzed to ensure > 80% B220+IgM+ naive B cells.
Gating strategy	The gating strategy is based on the live cell population and on the marker that has to be shown. A figure exemplifying the strategy is reported in the main or in the supplementary figures for every flow cytometry experiment performed.

☒ Tick this box to confirm that a figure exemplifying the gating strategy is provided in the Supplementary Information.
